# DNA methylation profiling for molecular classification of adult diffuse lower-grade gliomas

**DOI:** 10.1186/s13148-021-01085-7

**Published:** 2021-05-03

**Authors:** Sandra Ferreyra Vega, Thomas Olsson Bontell, Alba Corell, Anja Smits, Asgeir Store Jakola, Helena Carén

**Affiliations:** 1 Department of Clinical Neuroscience, Institute of Neuroscience and Physiology, Sahlgrenska Academy, University of Gothenburg, Gothenburg, Sweden; 2 Sahlgrenska Center for Cancer Research, Department of Laboratory Medicine, Institute of Biomedicine, Sahlgrenska Academy, University of Gothenburg, Gothenburg, Sweden; 3 Department of Physiology, Institute of Neuroscience and Physiology, Sahlgrenska Academy, University of Gothenburg, Gothenburg, Sweden; 4 Department of Clinical Pathology and Cytology, Sahlgrenska University Hospital, Gothenburg, Sweden; 5 Department of Neurosurgery, Sahlgrenska University Hospital, Gothenburg, Sweden; 6 Department of Neuroscience, Neurology, Uppsala University, Uppsala, Sweden; 7 Department of Neurosurgery, St. Olavs University Hospital, Trondheim, Norway

**Keywords:** DNA methylation profiling, Diffuse lower-grade glioma, DNA methylation-based classification, Molecular classification, Prognosis

## Abstract

**Background:**

DNA methylation profiling has facilitated and improved the classification of a wide variety of tumors of the central nervous system. In this study, we investigated the potential utility of DNA methylation profiling to achieve molecular diagnosis in adult primary diffuse lower-grade glioma (dLGG) according to WHO 2016 classification system. We also evaluated whether methylation profiling could provide improved molecular characterization and identify prognostic differences beyond the classical histological WHO grade together with *IDH* mutation status and 1p/19q codeletion status. All patients diagnosed with dLGG in the period 2007–2016 from the Västra Götaland region in Sweden were assessed for inclusion in the study.

**Results:**

A total of 166 dLGG cases were subjected for genome-wide DNA methylation analysis. Of these, 126 (76%) were assigned a defined diagnostic methylation class with a class prediction score ≥ 0.84 and subclass score ≥ 0.50. The assigned methylation classes were highly associated with their *IDH* mutation status and 1p/19q codeletion status. *IDH*-wildtype gliomas were further divided into subgroups with distinct molecular features.

**Conclusion:**

The stratification of the patients by methylation profiling was as effective as the integrated WHO 2016 molecular reclassification at predicting the clinical outcome of the patients. Our study shows that DNA methylation profiling is a reliable and robust approach for the classification of dLGG into molecular defined subgroups, providing accurate detection of molecular markers according to WHO 2016 classification.

**Supplementary Information:**

The online version contains supplementary material available at 10.1186/s13148-021-01085-7.

## Background

Diffuse lower-grade gliomas (dLGGs; World Health Organization [WHO] grade II and III) are primary infiltrative neoplasms of the central nervous system (CNS) that exhibits a highly variable clinical behavior and diverse biological features [1, 2]. These tumors recur frequently and will eventually undergo malignant transformation to gliomas of higher grades, worsening the prognosis of the patients [2–4].

For many years, the classification of diffuse gliomas relied purely on histopathological criteria and was subjected to high inter-observer variability, with substantial inconsistency in predicting clinical outcomes [1–5]. Major recent advances in genomic analysis have expanded our understanding of the molecular alterations characterizing dLGG, identifying central molecular biomarkers with diagnostic and prognostic capabilities. In the revised WHO 2016 classification, the incorporation of molecular biomarkers, such as mutations in the isocitrate dehydrogenase (*IDH*) genes 1 and 2 and codeletion of the 1p and 19q chromosomal arms (1p/19q codeletion), together with histological features, offers a more objective clinical prognostic stratification of patients with dLGG [6–8].

DNA methylation is the most extensively studied epigenetic mechanism, as it plays a key role in the regulation of gene expression and in the development of cells. Aberrant alterations of the methylome are found in several human diseases, including cancer [9, 10]. In recent years, genome-wide DNA methylation profiling has emerged as a powerful analytical tool for characterization of a wide variety of CNS tumors and has been shown to be a highly robust and reproducible technique for profiling fresh-frozen tumor samples and tumors archived as formalin-fixed paraffin-embedded (FFPE) samples [11, 12]. Furthermore, the use of DNA methylation profiling in the classification of CNS tumors, has been recognized to be a valuable asset to stratify patients into clinically relevant subgroups [13–16] and to facilitate an integrated diagnosis when diagnostic discrepancies are encountered [17–19].

In this study, we investigated the value of using DNA methylation profiling in adult patients with dLGG. Through generation of genome-wide methylation profiles from 166 tumor specimens, we retrospectively assessed the capacity of methylation profiling to achieve WHO 2016 classification directly and whether methylation profiling could add any useful molecular refinement to the WHO 2016 classification. We further evaluated the methylation-based classification in survival analyses for outcome prediction and compared this with the classical WHO grading in addition to *IDH* and 1p/19q codeletion status. We demonstrate that methylation profiling is a valuable technique for providing reliable diagnostic and prognostic information for patients with dLGG.

## Results

### Cohort characteristics

Between 2007 and 2016, a total of 210 adult patients underwent surgical resection for primary dLGG at the neurosurgical department at the Sahlgrenska University Hospital (Gothenburg, Sweden). Of all cases, 168 patients were subjected for methylation profiling, were we excluded two patients due to poor tumor quality after quality assessment of the methylation array data. The clinical characteristics of the included 166 patients are listed in Table 1.

**Table 1:** Clinical characteristics of the studied population with diffuse lower-grade gliomas (n = 166)

Variables	Lower-grade gliomas (%)	
	Grade II, n = 74	Grade III, n = 92
Gender		
Female	26 (35)	40 (43)
Ratio male:female	1.9	1.3
Age at diagnosis—years		
Mean ± SD	48 ± 13.5	41 ± 13.6
Age at diagnosis—groups		
18–29	7 (9)	16 (17)
30–39	17 (23)	24 (26)
40–49	17 (23)	23 (25)
50–59	16 (22)	21 (23)
60–69	15 (20)	6 (7)
≥ 70	2 (3)	2 (2)
Tumor location		
Frontal	40 (54)	51 (55)
Insular	3 (4)	3 (3)
Occipital	1 (1)	2 (2)
Parietal	6 (8)	11 (12)
Temporal	24 (32)	24 (26)
Basal ganglia	0	1 (1)
Type of surgery		
Biopsy	6 (8)	2 (2)
Resection	68 (92)	90 (98)
Size of tumor		
< 4 cm	17 (23)	14 (15)
4–6 cm	31 (42)	49 (53)
> 6 cm	26 (35)	29 (32)
Histopathological diagnosis (WHO 2007)		
Astrocytoma	45 (61)	46 (50)
Oligoastrocytoma	15 (20)	30 (33)
Oligodendroglioma	14 (19)	16 (17)
Molecular biomarkers		
* IDH*-mutant	14 (19)	1 (1)
* IDH-*wildtype	2 (3)	4 (4)
1p/19q codeletion	18 (24)	14 (15)

SD, Standard deviation

### Molecular characterization and reclassification according to WHO 2016 CNS classification system

Assessment of the *IDH* mutation status and 1p/19q codeletion is central in current diagnostics of diffuse gliomas [1]. Therefore, we evaluated the robustness of methylation profiling in detecting these diagnostic biomarkers compared to the clinically used molecular techniques for such purpose, i.e., immunohistochemistry (IHC), next-generation sequencing (NGS), Sanger sequencing, fluorescent *in situ* hybridization (FISH) and multiplex ligation-dependent probe amplification (MLPA) (Table 2).

**Table 2:** Schematic overview of the diagnostic analyses performed for determining molecular biomarkers according to WHO 2016 classification

Molecular diagnostic biomarkers	Molecular techniques used in clinical diagnostics	Methylation profiling
Tumor grade	Histology	–
*IDH*^a^ mutation status	Immunohistochemistry	G-CIMP^c^
	Next-generation sequencing	
	Sanger sequencing	
1p/19q codel.^b^	Fluorescent in situ hybridization Multiplex ligation-dependent probe amplification	Copy number variation profiles

^a^*IDH*, isocitrate dehydrogenase gene family

^b^Codel., complete codeletion of 1p/19q chromosomal arms

^c^Glioma CpG island methylator phenotype

Determination of *IDH* mutation status by Sanger sequencing analyses of 165 of the cases detected 120 tumors harboring *IDH* mutations. The *IDH* mutation-associated G-CIMP phenotype, inferred from the DNA methylation array data, showed 100% sensitivity and specificity when compared to Sanger sequencing. After re-examination of the dLGG cases, two cases showed discrepant results compared to IHC. The discrepant cases were re-analyzed by tissue microarray in combination with immunohistochemistry for the *IDH* mutation R132H and *ATRX* mutation analysis, which validated the results from Sanger sequencing/methylation profiling.

**Fig. 1:**
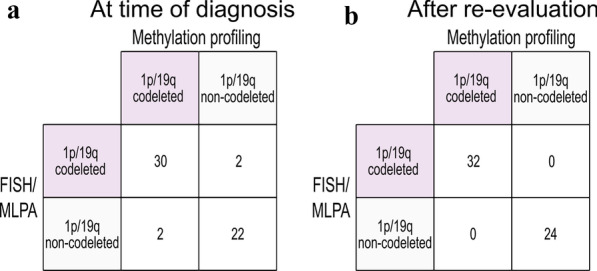
DNA methylation profiling for detection of diagnostic biomarkers. 1p/19q codeletion (n = 56) status was accurately detected by methylation profiling confirmed by molecular techniques used in clinical diagnosis [fluorescent in situ hybridization (FISH) and/or multiplex ligation-dependent probe amplification (MLPA)]. Left: comparison of methylation profiling with FISH/MLPA data at time of diagnosis, and right: after re-evaluation of the discrepant cases (n = 4)

Of 59 dLGG cases with clinical determination of 1p/19q codeletion status we excluded three cases from the analysis due to suspicion of normal brain tissue after evaluation of their CNV profiles generated from the methylation array. Of the included 56 cases, 32 cases presented 1p/19q codeletions. From the CNV profiles, we identified 1p/19q codeletions in 32 out of the 57 cases with a sensitivity of 94% and a specificity of 92% (Fig. 1). The samples with discrepant results (n = 4) were re-evaluated by a specialist in clinical neuropathology (TOB). For one of the four discrepant cases, re-examination revealed a misinterpretation of the FISH result at time of diagnosis and the case did not harbor a 1p/19q codeletion, corroborating the results from the methylation analysis. Re-evaluation of the remaining three discrepant cases, including additional clinical, radiological, histological and molecular data, supported the results from methylation analysis, increasing the sensitivity and specificity of the methylation array to 100%.

We next reclassified the dLGG cases by integrating molecular data (*IDH* mutation status and 1p/19q codeletion status) from IHC, NGS, Sanger sequencing, FISH and MLPA into the histopathological diagnosis. As our results showed that DNA methylation profiling provides accurate information of 1p/19q codeletion status (Fig. 1), we also incorporated 1p/19q codeletion status from CNV profiles to those cases where 1p/19q codeletion was not analyzed at time of clinical diagnosis (n = 107). Molecular reclassification of the dLGG cases according to the WHO 2016 criteria is shown in Additional file 1: Supplementary figure S1.

### DNA methylation-based classification

**Fig. 2:**
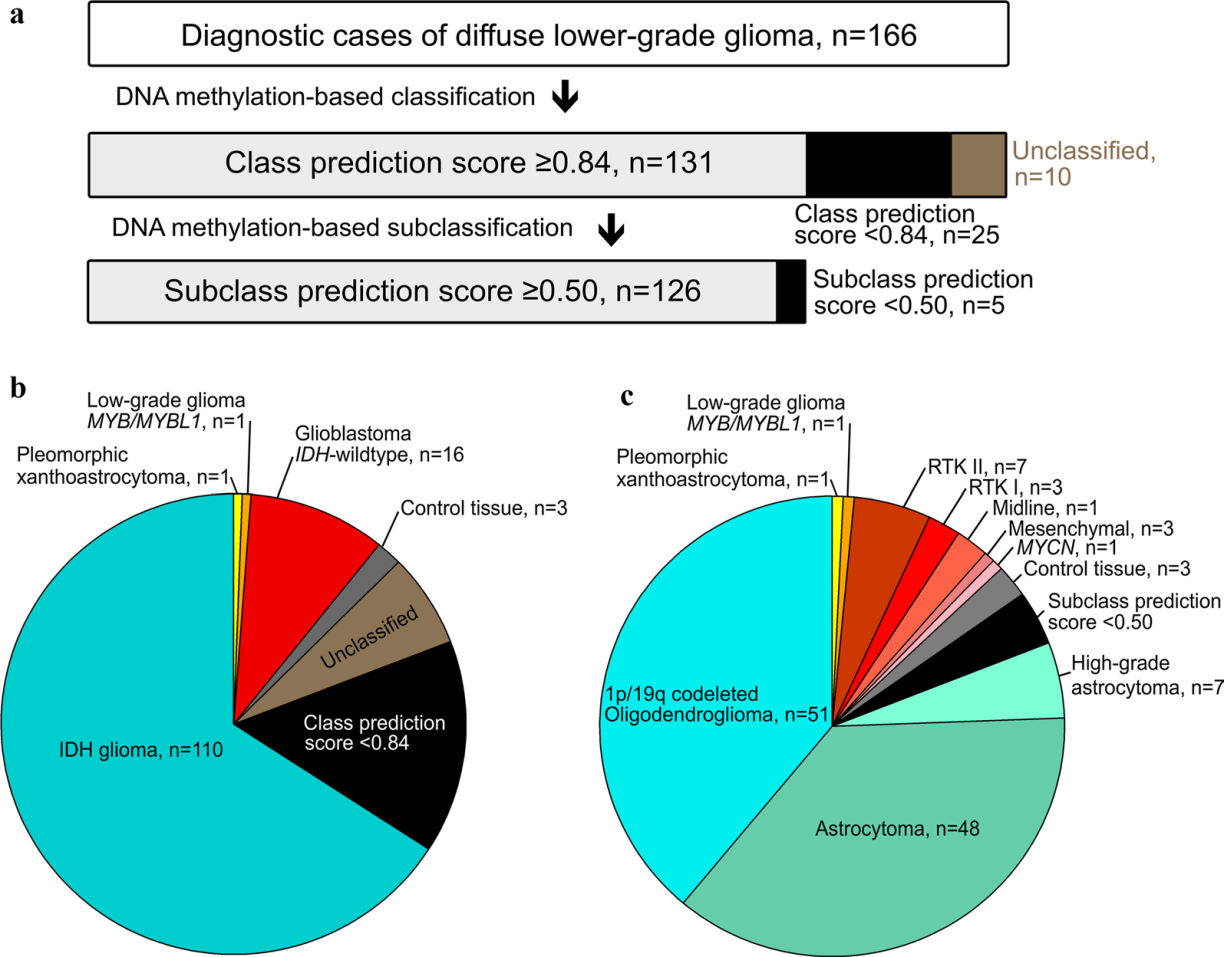
Overview of the adult diffuse lower-grade glioma (dLGG) classification based on methylation profiling. **a** Of the 166 profiled cases, 126 tumors were classified with a high class prediction score ≥ 0.84 and subclass prediction score ≥ 0.50 using the MNP classifier [13]. **b** Different methylation classes (n = 131) and **c** subclasses (n = 126) were identified in the glioma cohort using the MNP classifier [13]

DNA methylation profiles from the dLGG samples were analyzed by a DNA methylation-based classifier [13]. Of the 166 profiled cases, 79% (131/166) were assigned a defined DNA methylation class by the classifier tool with a class prediction score ≥ 0.84 (Fig. 2a). For 96% (126/131) of those cases, the classifier assigned a specific methylation subclass with a prediction score ≥ 0.50. The classifier was not able to predict a methylation class with a prediction score > 0.30, herein denoted as “unclassified” cases. This outcome was observed in 6% (10/166) of the cases and will be discussed further below.

We identified four distinctive methylation classes of brain tumors in the dLGG cohort with a class prediction score ≥ 0.84 (Fig. 2b). The majority of the cases were classified as glioma *IDH-*mutant (84%, 110/131), while 12% (16/131) were classified as glioblastoma *IDH*-wildtype. In addition, 2% (2/131) of the cases were classified as other tumor types than diffuse glioma, i.e., low-grade glioma *MYB*/*MYBL1* and (anaplastic) pleomorphic xanthoastrocytoma.

The methylation class glioma *IDH-*mutant comprised 48% (51/106) 1p/19q codeleted oligodendroglioma tumors, 45% (48/106) astrocytoma and 7% (7/106) high-grade astrocytoma (Fig. 2c). Within the glioblastoma *IDH*-wildtype methylation class, we found five methylation subclasses of which the methylation subclass receptor tyrosine kinase II (RTK II) represented the majority of the cases (47%, 7/15), followed by the 20% (3/15) RTK I and mesenchymal 20% (3/15).

**Fig. 3:**
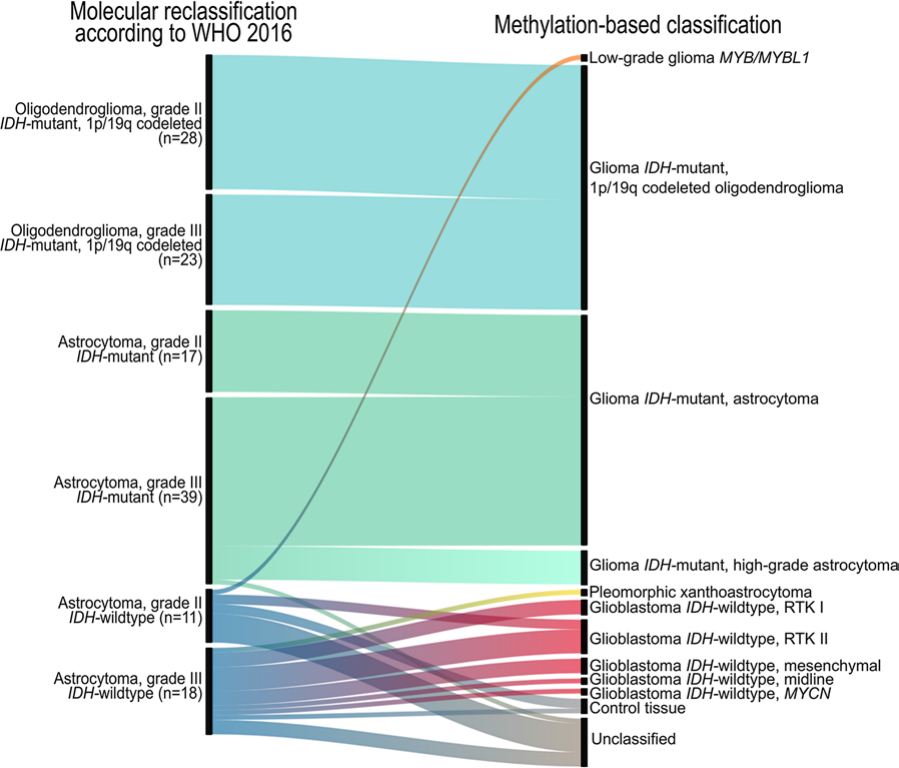
Molecular reclassification of the adult diffuse lower-grade glioma cohort according to WHO 2016 classification system. Associations of the molecular reclassification including WHO grading and molecular data (*IDH* mutation status and 1p/19q codeletions) generated at time of diagnosis and retrospectively in the study (left) with the outcome of methylation-based classification (right) with the MNP classifier [13]

In order to investigate the diagnostic value of methylation profiling, we compared the WHO 2016 molecular reclassification with methylation-based classification. Methylation profiling provided similar molecular characterization of dLGG cases as the integrated molecular diagnosis (Fig. 3). dLGG cases reclassified as oligodendrogliomas, harboring *IDH* mutations and 1p/19q codeletions, fall into the same methylation subgroup independently of the histopathological grade. *IDH*-mutant astrocytic gliomas were defined into two different methylation subgroups (astrocytoma or high-grade astrocytoma) that did not fully correspond to the assigned WHO grade. *IDH*-wildtype astrocytomas were the most heterogeneous molecular group as the grade II and grade III tumors were stratified into distinct methylation subclasses of glioblastoma or different molecular entities (i.e., low-grade glioma *MYB/MYBL1* or (anaplastic) pleomorphic xanthoastrocytoma) that are not included in the category of “diffuse astrocytic and oligodendroglial tumors” in the WHO 2016 classification system. The *IDH*-wildtype astrocytic gliomas classified as glioblastoma *IDH*-wildtype (n = 15) harbored other features characteristic of glioblastoma [20]. These included *EGFR* amplifications (87%, 13/15), whole chromosome 7 gain (53%, 8/15) or whole chromosome 10 loss (73%, 11/15). The *IDH*-wildtype astrocytic glioma classified as low-grade glioma *MYB/MYBL1* by methylation profiling, was negative for *IDH1/IDH2* and *BRAF* mutations by sequencing analysis and was 1p/19q non-codeleted. The diagnosis was changed to angiocentric glioma grade I (WHO 2016) after re-evaluation by the neuropathologist. Likewise, the *IDH*-wildtype astrocytoma classified as (anaplastic) pleomorphic xanthoastrocytoma harbored a *BRAF* V600E mutation and was *IDH1*-wildtype detected by Sanger sequencing. In addition, CNV analysis showed focal deletions of *CDKN2A/B* and no other chromosomal aberrations consistent with *IDH*-wildtype glioblastomas were found. This case was reevaluated and the diagnosis changed to anaplastic pleomorphic xanthoastrocytoma (WHO 2016).

### DNA methylation profiling for prediction of overall survival

**Fig. 4:**
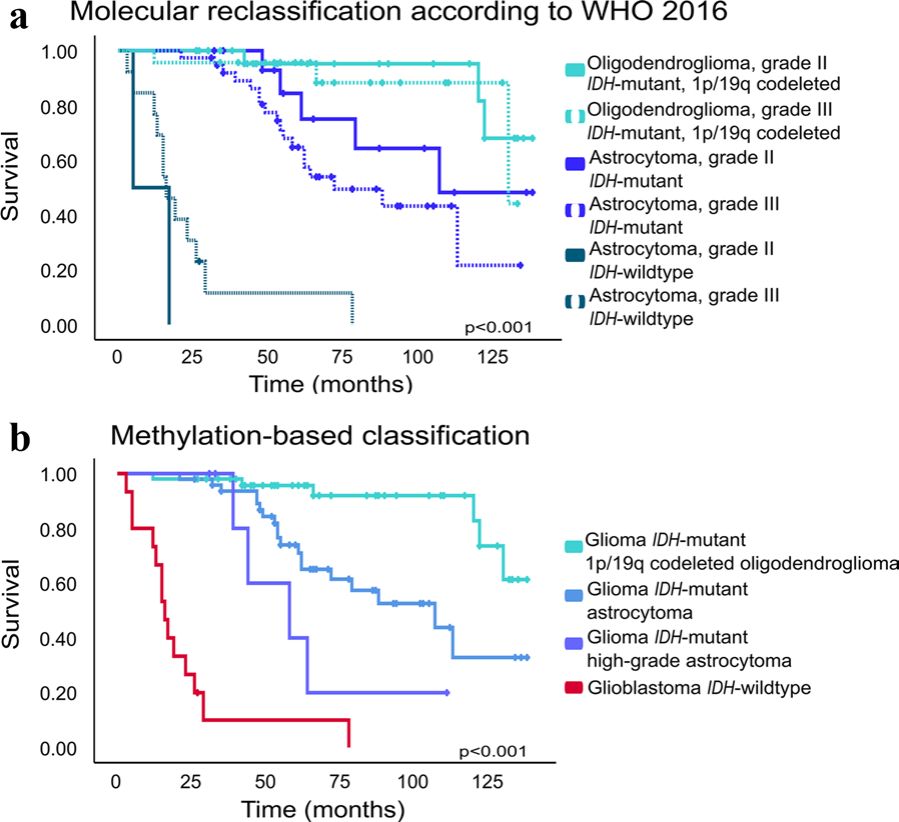
Kaplan–Meier overall survival analysis of 122 patients in the adult diffuse lower-grade glioma cohort. **a** Overall survival by molecular reclassification with *IDH* mutation status and 1p/19q codeletion status generated at time of diagnosis and retrospectively in the study. **b** Overall survival by methylation-based classification. The crossing bars on the lines for each survival curve represents censored information

We further investigated the potential of methylation profiling in predicting prognosis of the dLGG patients compared with the WHO 2016 molecular reclassification. Patients with tumors classified as control tissues by the classifier tool and entities with n = 1 were excluded from this survival analysis. Methylation-based classification provided similar prognostication compared to molecular reclassification using *IDH* mutation status and 1p/19q codeletion status according to WHO 2016 (Fig. 4a, b). Among the glioma *IDH*-mutant group, 1p/19q codeleted oligodendroglioma had a better prognosis (median survival not reached) than did patients with an astrocytoma subclass (median survival 115 months), who in turn had better prognosis than patients with high-grade astrocytoma subclass (median survival 60 months).

### *MGMT* promoter methylation and *CDKN2A/B* homozygous deletion as prognostic biomarkers in dLGG

The *MGMT* promoter methylation status is a predictive biomarker that can be used for treatment allocation in patients with glioblastoma [21, 22]. It has also been demonstrated to be an independent prognostic biomarker for high-risk low-grade glioma patients who received radiotherapy (RT) with adjuvant temozolomide (TMZ) treatment [23]. In our cohort, the *MGMT* promotor was methylated in 92% (97/106) and unmethylated in 8% (9/106) of the tumors classified as glioma *IDH*-mutant (Table 3). Of the 97 patients with a methylated promotor, 74% (72/97) received chemotherapy with TMZ and/or procarbazine, lomustine and vincristine. In the unmethylated group, six patients received such treatment. In the *IDH*-wildtype glioma group (i.e., glioblastoma, low-grade glioma *MYB/MYBL1* and (anaplastic) pleomorphic xanthoastrocytoma), *MGMT* promotor was methylated in 29% (5/17) and unmethylated in 71% (12/17) of the cases. Four patients in the methylated group were treated with alkylating agents compared to nine patients in the unmethylated group.

**Table 3:** Diagnostics by DNA methylation profiling of diffuse lower-grade gliomas with assigned methylation classes and subclasses (n = 126)

Methylation class	Molecular biomarkers according to methylation profiling				
	*IDH*^a^ wt (n = 20)	*IDH* mut (n = 106)	Codel.^b^ (n = 51)	*MGMT*^c^ unmeth (n = 24)	*MGMT* meth (n = 102)
*IDH* glioma (n = 106)					
1p/19q codeleted oligodendroglioma	0	51	51	0	51
Astrocytoma	0	48	0	7	41
High-grade astrocytoma	0	7	0	2	5
Glioblastoma *IDH* wt (n = 15)					
Mesenchymal	3	0	0	3	0
Midline	1	0	0	0	1
* MYCN*	1	0	0	1	0
RTK I	3	0	0	2	1
RTK II	7	0	0	5	2
Pleomorphic xanthoastrocytoma	1	0	0	0	1
Low-grade glioma *MYB/MYBL1*	1	0	0	1	0
Control tissue	3	0	0	3	0

^a^*IDH*, isocitrate dehydrogenase gene family, wildtype (wt) and mutated (mut)

^b^Codel., complete codeletion of 1p/19q chromosomal arms

^c^*MGMT*, O^6^-methylguanine-DNA methyltransferase promotor, methylated (meth) or unmethylated (unmeth)

Homozygous deletion of the *CDKN2A/B* gene has been identified as a biomarker for improved grading of *IDH*-mutant diffuse astrocytic gliomas [24]. We therefore studied the prognostic value of *CDKN2A/B* in our classified tumor cohort (n = 123). Homozygous deletion of the *CDKN2A/B* gene was detected in a larger proportion of the glioblastoma *IDH*-wildtype tumors (9/15) compared to glioma *IDH*-mutant tumors (9/106) (Fisher’s exact test *p* value < 0.001). Survival analysis did not show significant differences according to *CDKN2A/B* status within the defined molecular subgroups, possibly due to the limited number of cases.

### Evaluation of dLGG cases with low classification scores and unclassified cases

**Fig. 5:**
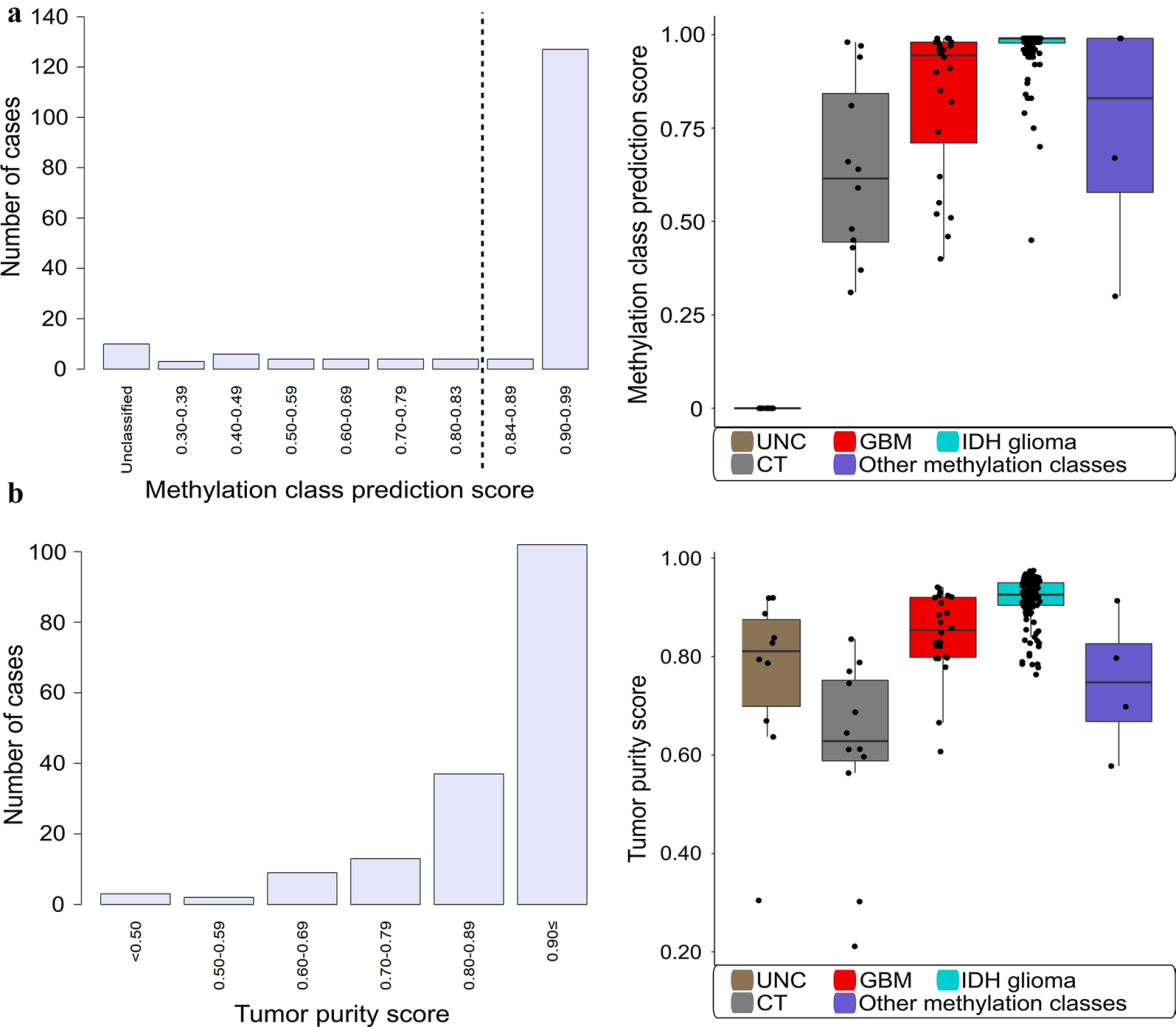
DNA methylation-based classification and tumor purity evaluation. **a** Left: distribution of the adult diffuse lower-grade glioma (dLGG) cases (n = 166) by methylation class prediction score. The vertical dotted line shows the threshold value of ≥ 0.84 for assignment to an established methylation class. Right: median methylation class score values by methylation classes. Black dots represent individual tumor cases in each methylation class. **b** Left: distribution of dLGG cases (n = 166) by tumor purity score estimated with InfiniumPurify [27, 28]. Right: median tumor purity score values by methylation classes (n = 166). IDH glioma: glioma *IDH*-mutant. GBM: glioblastoma *IDH*-wildtype. CT: control tissue. UNC: unclassified (non-classifiable cases with a class prediction score threshold ≥ 0.30). Other methylation classes: low-grade glioma, *MYB/MYBL1*, (anaplastic) pleomorphic xanthoastrocytoma, low-grade glioma, ganglioglioma and plexus tumor, pediatric B

To fully substantiate the impact of methylation-based classification for the WHO 2016 classification system, we evaluated the cases with low class/subclass prediction scores as these classification results can still provide important diagnostic information [25]. Three percent (5/166) of the tumors were assigned a methylation class (class prediction score ≥ 0.84) but were not further subclassified (subclass prediction score < 0.50), and for 21% (35/166), the class prediction score ranged between 0.30 and 0.83 (Fig. 2a and 5a) or were unclassified. The median methylation class score notably varied across the methylation classes (Fig. 5a). Methylation-based classification suggested, in the majority of the cases, diagnoses supported by CNV signatures and prognostic outcomes of the predicted methylation class/subclass (Additional file 1: Figure S2A–B). For example, the molecular reclassification of *IDH*-mutant, 1p/19q codeleted oligodendrogliomas (n = 3) was in accordance with the given methylation subgroup. *IDH*-wildtype astrocytic gliomas with a glioblastoma classification (n = 9), presented typical chromosomal changes (*EGFR* amplification, chromosome 7 gain or chromosome 10 loss) and prognosis of glioblastoma with the exception of the case classified as glioblastoma *IDH*-wildtype, midline (Additional file 2: table S1). For other *IDH*-wildtype astrocytic gliomas, particularly of grade II, the suggested methylation class was not associated with dLGG. For one of these cases, the methylation class plexus tumor, subclass pediatric B was predicted with a score of 0.30 by the classifier and the CNV profile from the methylation array was not indicative of a plexus tumor [26]. Other tumors were classified with the brain control tissue methylation classes (hemispheric cortex, hypothalamus, white matter or inflammatory tumor microenvironment) with prediction scores varying between 0.31 and 0.81. Tumor CNV profiles were identified in 44% (4/9) of these tumors.

For better interpretation of the cases with low classification prediction scores and unclassified cases, we accounted for tumor purity as low neoplastic cell content could influence the classification of the tumors [13]. As the proportion of neoplastic cells was not assessed prior to running the samples in the methylation array, we used the R package InfiniumPurify [27, 28] to estimate tumor purity. Tumor purity ranged from 21 to 97% (Fig. 5b) in the dLGG cohort. Among the cases with low prediction scores, 30% (9/30) showed a tumor purity of 70% or lower (Additional file 1: Figure S2B). The median tumor purity score was quite high among the methylation classes except for the control tissue class with an estimated median tumor purity score below 70% (Fig. 5b). In some cases, the low methylation class predicted score could be explained by the low neoplastic cell content but not all (Additional file 1: Figure S2C). Using a tumor purity cutoff of ≥ 70% as suggested by Capper et al*.* [25], 152 cases remain and 10% (15/152) of these have a low class prediction score. However, the low number of neoplastic cells could still provide a valid classification in some cases.

## Discussion

Genome-wide DNA methylation characterization of CNS tumors has facilitated the classification of tumors into molecularly defined subgroups with distinct clinical prognoses and outcomes [13–16]. Several institutional centers have started to evaluate the impact of DNA methylation analysis toward its implementation into clinical practice [13, 17, 18, 29, 30]. The value of methylation profiling in the diagnosis and prognosis of primary dLGG has however not been investigated. We therefore generated methylation data from 166 dLGG tumors, assessed the impact of methylation profiling on clinical molecular diagnostics compared to the WHO 2016 classification system and evaluated its prognostic utility for clinical outcomes.

Molecular classification with DNA methylation analysis was achieved for 76% of the dLGG cases using the MNP classifier [13]. Previous studies have shown classification rates of 49–95% [13, 17, 18, 30]. These differences can be explained by the different cohorts and selection of patients that have been included in the different studies. For example, in the study by Jaunmuktane et al*.* [17] the authors reported a match in 56% of their adult brain tumor cases with a calibrated score ≥ 0.84. The higher classification rate in our cohort using the same cutoff value, could be explained by the population-based setup of our study, rather than examining a mixed cohort of patients with, e.g., diagnostically challenging cases or cases referred for second opinion.

The molecular classes identified in our cohort were strongly related to the *IDH* mutation- associated G-CIMP status verified by Sanger sequencing analysis. The clinical analysis of *IDH* mutation status by IHC yielded two discrepant results compared to Sanger sequencing/methylation profiling, warranting some cautions in the interpretation of IHC analysis alone. Likewise, 1p/19q codeletions were detected with high accuracy by analyzing the generated CNV profiles, which were confirmed by the clinical analysis (FISH and/or MLPA). This demonstrates that methylation analysis provides reliable detection of *IDH* mutations and 1p/19q codeletions and can further resolve inconclusive chromosomal aberrations that could lead to misinterpretation.

The diagnosis and prognostication provided by the methylation analysis showed good concordance with the currently used WHO 2016 classification system, demonstrating that it is a valid method for routine classification. Furthermore, it provides more comprehensive data, allowing detection of rare tumor entities that are easily to be misclassified using conventional techniques, in addition to further identify the more indolent *IDH-*wildtype gliomas from molecular glioblastoma without the need for additional analyses.

Promotor methylation of the *MGMT* gene is a strong predictor of therapy response and survival in patients with glioblastoma [21, 22] and has been suggested as a prognostic biomarker for high-risk dLGG treated with RT and TMZ [23]. We cannot draw any conclusions regarding the predictive value of *MGMT* in a cohort of dLGG. Thus, further studies with larger cohorts are required to validate the value of *MGMT* promotor methylation in dLGG.

Homozygous deletion of the chromosomal region harboring the *CDKN2A* and *CDKN2B* genes was recently proposed as a prognostic biomarker for diffuse gliomas [24]. We assessed its prognostic value in our cohort, but the cohort contained too few dLGG cases with homozygous *CDKN2A/B* deletions to provide a valid comparison between the groups. Shirahata et al. [24] identified 18% deletions in 211 *IDH*-mutant astrocytic gliomas, where all deletions were detected in grade III astrocytomas and glioblastomas. In our study, we detected 8% deletions in 106 *IDH*-mutant gliomas using the same cutoff value. Our *IDH-*mutant glioma cohort comprised approximately 50% astrocytic tumors with only a minority subclassified as high-grade gliomas, which explains the low number of deletions in the cohort.

Tumor specimens are heterogeneous mixtures of healthy normal cells and neoplastic cells. Hence, estimates of tumor purity should be taken into consideration when interpreting methylation-based classification of tumors [13]. In our study, we did not set a cutoff value for tumor purity but examined how methylation profiling would work in all dLGG cases regardless of the percentage of neoplastic cells in the tumors. Some of the unclassified cases showed a low percentage of neoplastic cells. However, the majority of the unclassified cases showed a tumor purity above 70%, suggesting that these cases may represent rare tumor entities that are not included in the MNP classifier.

A limitation of this study is the lack of molecular data for all patients at time of diagnosis. The patients in our cohort were diagnosed with dLGG in the era where molecular markers were not required to establish a diagnosis. In addition, we did not conduct prospective analyses, which are needed to elucidate the impact of methylation profiling on patients with changed treatment approach when integrating diagnostic information provided by methylation analysis.

## Conclusions

In conclusion, our results substantiate the value of DNA methylation profiling to diagnose and distinguish different dLGG entities, as well as to predict prognosis of the patients. The identification of diagnostic and prognostic biomarkers in a robust high-throughput fashion is feasible through methylation arrays. Relevant glioma diagnostic and prognostic biomarkers such as *IDH* mutation status, copy number aberrations including 1p/19q codeletions, *MGMT* promotor methylation status and differentiation of *IDH*-wildtype tumors into glioblastoma or indolent tumors, can all simultaneously be obtained from one analysis. This may facilitate the current routine molecular diagnostic approaches by replacing multiple analyses for the different biomarkers with one assay.

## Methods

### Patients and samples

A clinical consecutive cohort of 210 adult patients, diagnosed with primary dLGG (WHO 2007 grade II-III) during 2007–2016 in the Västra Götaland region in Sweden, was evaluated for inclusion. Patients with suspected radiological appearance of a glioblastoma (e.g., ring-like contrast enhancement and necrosis) from which only a diagnostic biopsy was taken, but where biopsy results were a grade II or III glioma, were not included due to the risk of sampling bias [31, 32]. This was the selection criterion for a larger dLGG study, where methylation-based re-analysis was one of the research aims. We also excluded patients with non-available FFPE tumor tissue for further analyses. FFPE tumor tissue and clinical data (patient characteristics and molecular data) were collected retrospectively for a total of 168 subjects. Molecular data included *IDH* mutation status determined for 21 dLGG cases (15 *IDH*-mutant and 6 *IDH*-wildtype cases) by IHC and/or NGS and 1p/19q codeletion status for 60 dLGG cases (56 1p/19q codeleted cases) by FISH and/or MLPA determined at time of diagnosis.

### Genome-wide DNA methylation analysis

#### DNA extraction and quantification

DNA from FFPE tumors was extracted with QIAamp® DNA FFPE kit (Qiagen) following the protocol provided by the manufacturer with an extra digestion step with proteinase K overnight. The extracted DNA was quantified using the Qubit® dsDNA High Sensitivity Assay Kit (Thermo Fisher Scientific).

#### Bisulfite conversion of DNA, restoration and array processing

Between 500 and 1000 ng of extracted DNA was bisulfite-converted with the EZ DNA methylation kit (Zymo) and restored with the Infinium HD FFPE Restore Kit (Illumina), according to the instructions supplied by the manufacturer. The Infinium MethylationEPIC BeadChip array (Illumina) was used to generate genome-wide DNA methylation profiles for the tumor samples (UCL Genomics, UK).

#### Data analysis

Raw methylation data (IDAT files) generated from the methylation arrays were normalized, analyzed and assessed for quality controls as previously described [33] using the statistical software R with Rstudio (version 4.0.2). The G-CIMP phenotype, which is strongly associated with *IDH* mutations in gliomas [25, 34, 35], was characterized by unsupervised hierarchical clustering to identify dLGG samples with *IDH* mutations. Hierarchical clustering was performed with the 1500 most deviating CpG sites with The Cancer Genome Atlas glioma samples with known G-CIMP status based on methylation data as described by Noushmehr et al.[35]. The O^6^-methylguanine-DNA methyltransferase (MGMT) promotor methylation status was predicted with the R package MGMT-STP27 [37] using the suggested cutoff at 0.358 [36, 37]. Percentage of neoplastic cells was determined with the R package InfiniumPurify [27, 28] with normal methylation data included in the package. For tumor classification, IDAT files were uploaded into a publicly available DNA methylation-based classifier (MNP, version 114b, https://www.molecularneuropathology.org/mnp) [13]. Methylation-based tumor classifications and subclassifications with prediction class scores, indicating probability estimations of the assigned classes, were automatically generated as reports by the classifier tool. The reports were then evaluated according to the recommendations as presented by Capper et al. [25] using the threshold value ≥ 0.84 preferred for clinical settings.

#### Copy number variation analysis

Copy number variation (CNV) profiles for each individual case were generated from raw methylation data inferred from the methylation array using the R package conumee [38]. 1p/19q codeletion, homozygous deletion of *CDKN2A/B*, amplification of the epidermal growth factor receptor (*EGFR*) as well as gain of chromosome 7 and loss of chromosome 10, were visually assessed from the CNV profiles. For determining *CDKN2A/B* deletion we used the cutoff previously suggested by Shirahata et al*.* [24].

### Mutation analysis of *IDH1/2* and *BRAF* by Sanger sequencing

PCR amplification of *IDH1, IDH2* and *BRAF* was performed with 50 ng of extracted DNA using *IDH1*, *IDH2* [39] or *BRAF* primers. PCR products were purified with ExoSAP-IT PCR Product Cleanup Reagent (Affymetrix) before Sanger sequencing (Eurofins—GATC Biotech).

### Molecular reclassification

Molecular reclassification of the dLGG cases following the WHO 2016 criteria was based on histopathological grade and *IDH* mutation status, 1p/19q codeletion status and, in some instances alpha thalassemia/mental retardation syndrome X-linked (*ATRX*) status, generated at time of diagnosis and retrospectively in our facilities.

### Statistical analysis

Statistical analyses were performed using the IBM SPSS® Statistics software version 25. We conducted a Kaplan–Meier survival analysis and overall survival curves (from date of surgery to date of death or date of last follow-up) were compared with a log-rank test.

## Supplementary Information

**Additional file 1:** Figure S1. This figure presents the molecular reclassification of the adult diffuse lower-grade glioma cohort according to WHO 2016. Supplementary figure S2. This figure presents diagnostic and prognostic evaluations of diffuse lower-grade glioma cases with low methylation class/subclass prediction scores and evaluation of tumor purity.

**Additional file 2**: Table S1. This table presents the clinical and molecular characteristics of diffuse lower-grade glioma cases with low methylation class/subclass prediction scores.

## Data Availability

Not applicable.

## Abbreviations

ATRX: Alpha thalassemia/mental retardation syndrome X-linked
CNS: Central nervous system
CNV: Copy number variation
dLGG: Diffuse lower-grade glioma
EGFR: Epidermal growth factor receptor
FFPE: Formalin-fixed paraffin-embedded
FISH: Fluorescent in situ hybridization
G-CIMP: Glioma CpG island methylator phenotype
IDH: Isocitrate dehydrogenase gene family
IHC: Immunohistochemistry
MGMT: O^6^-methylguanine-DNA methyltransferase promotor
MLPA: Multiplex ligation-probe amplification
NGS: Next-generation sequencing
RTK I/II: Receptor tyrosine kinase I or II
TMZ: Temozolomide
WHO: World Health Organization
