# Supplementary material for: DNA methylation profiling for molecular classification of adult diffuse lower-grade gliomas
Source: Clin Epigenetics. 2021 May 3;13:102. doi: 10.1186/s13148-021-01085-7 (PMC8091784; doi:10.1186/s13148-021-01085-7)
Supplement: Supplementary file 1 — Additional file 1: Figure S1. This figure presents the molecular reclassification of the adult diffuse lower-grade glioma cohort according to WHO 2016. Supplementary figure S2. This figure presents diagnostic and prognostic evaluations of diffuse lower-grade glioma cases with low methylation class/subclass prediction scores and evaluation of tumor purity. [file 13148_2021_1085_MOESM1_ESM.pdf]

Additional file 1: Supplementary figures

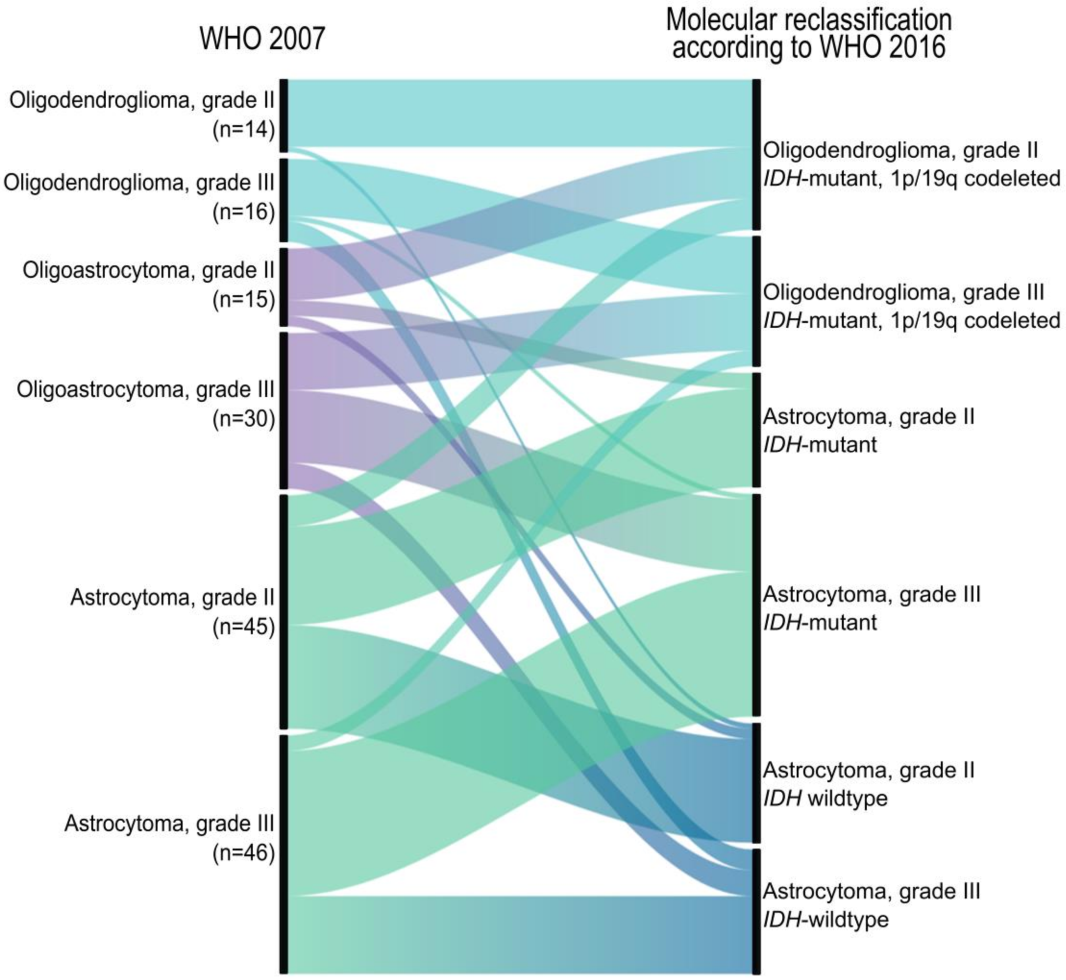

Supplementary figure S1. **Molecular reclassification of the adult diffuse lower-grade glioma cohort according to WHO 2016.** Association of the WHO 2007 histopathological diagnosis (left) with molecular reclassification (right) including WHO grade and molecular data (*IDH* mutation status and 1p/19q codeletion status) generated at time of diagnosis and acquired from Sanger sequencing and copy number variation profiles generated from the methylation array.

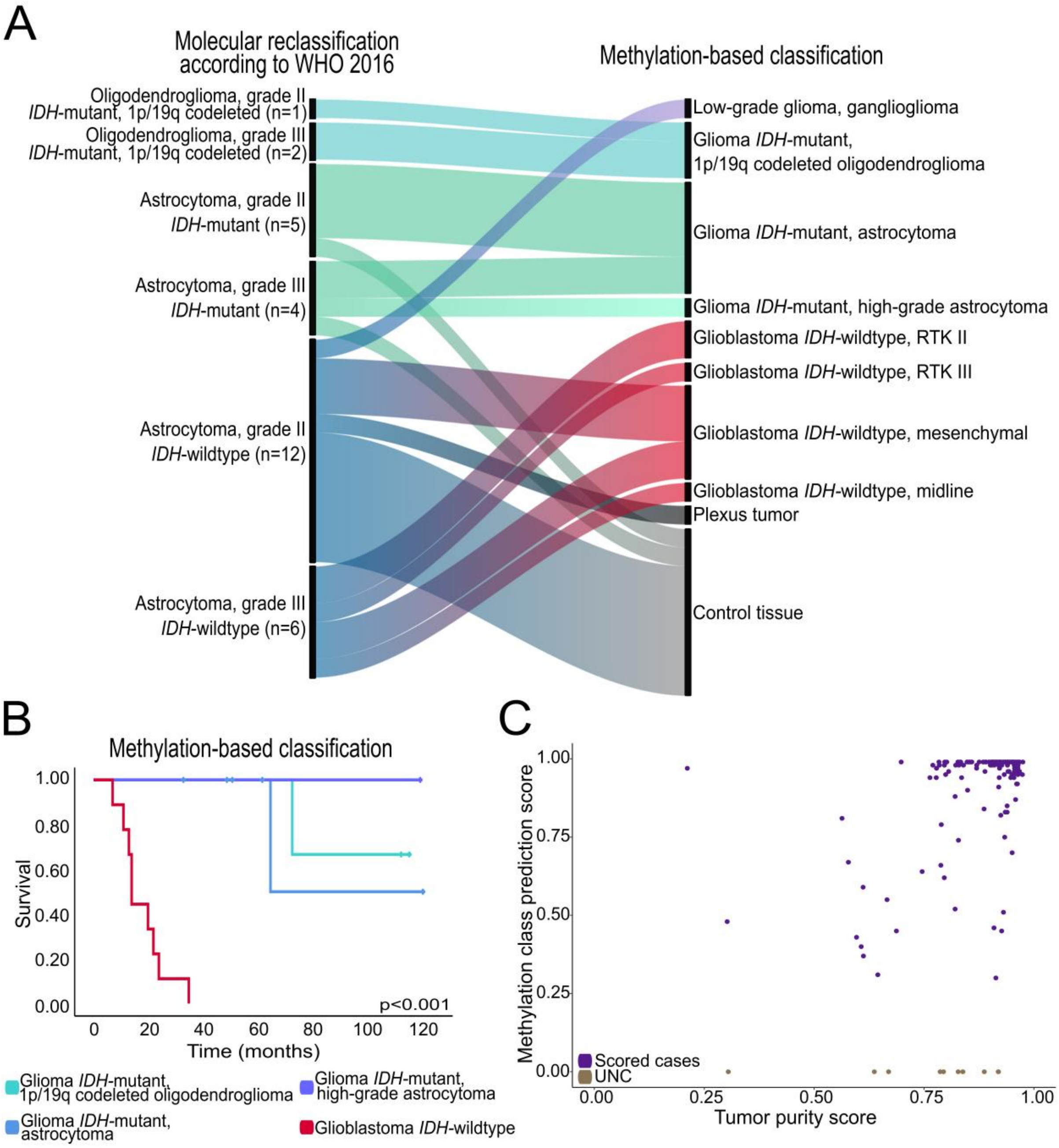

Supplementary figure S2. (A) Associations of the molecular WHO 2016 reclassification (left) with the outcome of methylation-based classification (right) for diffuse lower-grade glioma (dLGG) cases with low prediction scores. (B) Kaplan-Meier overall survival analysis of 19 patients in the dLGG cohort with tumors classified with low methylation class prediction scores. The crossing bars on the lines for each survival curve represents censored information. (C) Association between methylation class prediction score and the estimated tumor purity score with InfiniumPurify [1,2] for all dLGG cases.

REFERENCES

1. Qin Y, Feng H, Chen M, Wu H, Zheng X (2018) InfiniumPurify: An R package for estimating and accounting for tumor purity in cancer methylation research. Genes & diseases 5:43-45. doi:10.1016/j.gendis.2018.02.003

2. Zheng X, Zhang N, Wu HJ, Wu H (2017) Estimating and accounting for tumor purity in the analysis of DNA methylation data from cancer studies. Genome biology 18:17. doi:10.1186/s13059-016-1143-5
